# Branched-chain amino acids and the risk of hypertension; a persian cohort-based study

**DOI:** 10.1186/s12872-024-04045-0

**Published:** 2024-07-31

**Authors:** Zahra Salimi, Bahareh Aminnezhad Kavkani, Pooneh Allahyari, Seyed Ali Askarpour, Zahra Mahmoudi, Mahdie Torkaman, Mohadeseh Sadat Mousavi Hoseini, Zahra Mousavi, Shirin Tajadod, Neda Valisoltani, Sara Khoshdooz, Saeid Doaei, Akram Kooshki, Maryam Gholamalizadeh

**Affiliations:** 1 Student Research Committee, Faculty of Nutrition and Food Technology, Shahid Beheshti University of Medical Sciences, Tehran, Iran; 2 Department of Nutrition. Science and Research Branch, Islamic Azad University, Tehran, Iran; 3 Department of Exercise Physiology, Faculty of Physical Education and Sport Sciences, Central Tehran Branch, Islamic Azad University, Tehran, Iran; 4 Department of Environmental Health Engineering, Division in Food Safety and Hygiene, Tehran University of Medical Sciences, Tehran, Iran; 5 Department of Nutrition, Science and Research Branch , Islamic Azad University, Tehran, Iran; 6 Department of Chemical Engineering, Science and Research Branch, Islamic Azad University, Tehran, Iran; 7 Department of Food Science and Technology, Science and Research Branch, Islamic Azad University, Tehran, Iran; 8 Nursing and Midwifery school, Shahed University, Tehran, Iran; 9 Department of Nutrition, School of Public Health, International Campus, Iran University of Medical Sciences, Tehran, Iran; 10 Department of Clinical Nutrition, School of Nutrition Science and Dietetics, Tehran University of Medical Sciences, Tehran, Iran; 11 Faculty of Medicine, Guilan University of Medical Sciences, Rasht, Iran; 12 Department of Community Nutrition, School of Nutrition and Food Sciences, Shahid Beheshti University of Medical Sciences, Tehran, Iran; 13 Non-Communicable Diseases Research Center, Department of Nutrition & Biochemistry, School of Medicine, Sabzevar University of Medical Sciences, Sabzevar, Iran; 14 Cancer Research Center, Shahid Beheshti University of Medical Sciences, Tehran, Iran

**Keywords:** Amino acids, Branched-chain amino acids, Blood pressure, Hypertension

## Abstract

**Background:**

The association of hypertension (HTN) and different types of dietary amino acids is not yet clear. The aim of the present study was to investigate the association of branch chain amino acids (BCAAs) and the prevention of HTN.

**Methods:**

This cross-sectional study was conducted on 4184 people aged 35 to 70 using data from the Sabzevar Persian cohort study in Sabzevar, Iran. Data on dietary intake of BCAAs including leucine, isoleucine, and valine were obtained using a validated Food Frequency Questionnaire (FFQ). Multivariable logistic regression analysis assessed the link between HTN and BCAAs.

**Results:**

The results showed that participants with HTN had a higher total protein and BCAAs intake than participants with normal BP (*P* < 0.01). A marginally significant association was found between the risk of HTN with the total intake of BCAAs (OR = 1.018, CI95%: 1.001–1.035, *P* = 0.04), leucine (OR = 1.040, CI95%:1.002–1.080, *P* = 0.03), isoleucine (OR = 1.068, CI95%:1.001–1.140, *P* = 0.04), and valine (OR = 1.060, CI95%:1.003–1.121, *P* = 0.04). However, the association disappeared after adjusting the total protein and calorie intake.

**Conclusions:**

The results indicated that the dietary intake of BCAAs may be associated with the risk of HTN. Future longitudinal research is warranted.

## Introduction

Hypertension (HTN) is a chronic disease characterized by elevated blood pressure (BP) [1], which is typically the cause of numerous cardiovascular disorders such as coronary artery disease, stroke, and heart failure [2]. HTN affects more than one billion adults worldwide [3], and the global number of people living with HTN will reach two billion by 2025 [4]. The prevalence of hypertension among adults is higher in low and middle-income countries (LMICs), affecting 31.5% of the population (1.04 billion people), compared to high-income countries (HICs), where it affects 28.5% of the population (349 million people) [5]. According to the American College of Cardiology/American Heart Association (ACC/AHA), in 2017, approximately 46% of Americans have HTN [6]. In China, a significant increase was reported in the prevalence of HTN among adults aged 20 years and older, from 25.7% in 2007 to 31.5% in 2017 [7]. Based on findings of the population-based national surveys in Iran, the prevalence of HTN increased from 14.66% in 2009 to 32.03% in 2021 [5].

HTN is affected by many factors including lifestyle [8], overweight [9], and dietary components such as sodium [10]. Dietary modifications are considered a beneficial strategy in the treatment of HTN [11, 12]. The association between dietary proteins and HTN has recently received increased attention due to their potential role in cardiovascular function [13]. Dietary protein intake was reported to be associated with both systolic and diastolic BP [13, 14]. However, data regarding the possible effects of different types of dietary protein on HTN are inconsistent [14, 15].

BCAAs comprising leucine, isoleucine, and valine have an aliphatic side-chain with a branch, a unique structure that allows them to be metabolized primarily in the muscles rather than the liver, unlike other amino acids [16]. These amino acids are abundant in animal proteins such as meat, fish, dairy products, and eggs, as well as in legumes, which are plant sources of dietary protein. An adequate intake of BCAAs is required for body protein synthesis, production of energy, and several metabolic functions [17, 18], glucose metabolism, and regulation of some pivotal pathways [19, 20].

BCAAs have been previously reported to be inversely related to BP [21]. On the other hand, some other studies have suggested that higher levels of circulating BCAAs are positively associated with HTN [14, 22]. Alterations in BCAAs metabolism leading to aggregation of BCAAs and their byproducts are associated with significant metabolic disorders such as insulin resistance and an increased risk of HTN [23, 24]. In addition, BCAAs intake diminishes brain tyrosine and tryptophan uptake and may decrease catecholamine and serotonin synthesis, adversely affecting central BP regulation [25, 26].

Although many studies have examined the relationship between individual essential amino acids in the form of plant or animal protein categories with BP [27, 28], the association of different types of BCAAs with HTN is not yet clear. Considering the high prevalence of hypertension in adults worldwide [4] and to test whether the BCAAs could be positively and negatively related to BP, the present study was conducted to investigate the association of BCAAs with the risk of HTN.

## Methods

### Study population

This cross-sectional study was carried out on Iranian adults using Sabzevar Persian cohort data, which was performed from January 2017 to May 2020 on 4184 people including 1239 people with HTN and 2945 people without HTN in Sabzevar, Iran. Inclusion criteria included age between 35 and 70 years, no family history of HTN, no history of kidney diseases, no treatment with drugs affecting BP, and no history of BCAAs supplementation. Exclusion criteria included the inability to collect the required data.

### Data collection

The Persian cohort questionnaires collected data on general and socio-demographic factors, medical status, and dietary intake through interviews and physical examinations. The SECA 755 mechanical column scale and SECA 204 mobile stadiometer were used to measure weight and height without shoes in light clothes to the nearest 100 g and 0.5 cm, respectively. The participants’ BMI was then calculated as weight (kg)/height^2^ (m^2^) [29].

The Persian cohort food frequency questionnaire (FFQ) was used to evaluate dietary intake, and its validity and reliability have already been confirmed [30]. The collected data on dietary intake was assessed using the Nutritionist IV software regarding dietary calories, total protein, and BCAAs. In this study, the amount of food consumed by each person during the last year was assessed, and the total amount of BCAAs intake was estimated using the amount of BCAAs in each food item based on the USDA food database.

BP was measured in the morning after 10 min rest period twice in each arm supported at heart level in a seated position of the participants with their back supported and legs uncrossed in a quiet room after ten-minute intervals, using Richter auscultator mercury sphygmomanometers (MTM Munich, Germany) with appropriately adjusted cuff size [29]. The participants were advised to avoid coffee (and other sources of caffeine) before checking their BP. The average of the measurements was used in the analyses.

### Statistical analysis

The independent t-test or Mann-Whitney test (for non-parametric variables) was used to compare quantitative data, and the chi-square test was used to compare qualitative variables between the participants with and without HTN. Hypertension was defined as systolic blood pressure ≥ 140 mmHg and/or diastolic blood pressure ≥ 90 mmHg [31]. A logistic regression test was used to assess the association between HTN as a categorical variable and dietary intake of BCAAs as a continuous variable. The confounders were adjusted using different models of logistic regression including the crude model (Model 1), adjusted for age and gender (Model 2), further adjustments for education, marital status, job, physical activity, BMI, and diabetes (Model 3), and additional adjustments for the intake of energy, protein and other amino acids (Model 4). Kolmogorov-Smirnov test assessed the normal distribution of data, and all analyses were performed using SPSS version 21, considering the significant level at *P* < 0.05.

## Results

In total, 4184 people including 2945 people with normal BP and 1239 with HTN participated in the study. The participants with HTN had higher age (53.45 ± 8.3 vs. 47.46 ± 8.35 y), height (163.32 ± 9. 5 vs. 161.75 ± 9.1 cm), weight (78.01 ± 14. 2 vs. 72.51 ± 12.8 kg), body mass index (BMI) (29.23 ± 4.7 vs. 27.76 ± 4. 7 kg/m^2^), right arm systolic BP (RSBP) (134.12 ± 13 vs. 106.23 ± 10. 6 mmHg), and right arm diastolic BP (RDBP) (All *P* < 0.01). Also, the patients with HTN had higher occupation and diabetes and lower education compared to the participants without HTN (Table 1).

**Table 1:** The characteristics of the participants

	SBP ≤ 140 and/or DBP ≤ 90 (*n* = 2945)	SBP > 140 and/or DBP > 90 (*n* = 1239)	P*
Age (y)	47.46 ± 8.35	53.45 ± 8.3	< 0.001
Gender			
Male N (%)	1166 (39.59%)	700 (56.50%)	0.1
Female N (%)	1779 (60.41%)	539 (43.5%)	
MET	38.84 ± 7.8	38.1 ± 7.7	0.008
Height (cm)	161.75 ± 9.1	163.32 ± 9.5	< 0.001
Weight (Kg)	72.51 ± 12.8	78.01 ± 14.2	< 0.001
BMI (Kg/m^2^)	27.76 ± 4.7	29.23 ± 4.7	< 0.001
Use Alcohol (n, %)	257 (8.74)	123 (9.94)	0.48
Married	2744 (93.2)	1167 (94.2)	0.07
Smoking (n, %)	731 (24.83)	238 (19.21)	0.06
Has Job n (%)	821 (27.9)	617 (49.8)	0.001
Education			
Diploma≥	1884 (64)	756 (61)	0.01
> Diploma	1061 (33)	483 (23)	
Has diabetes	315 (10.7%)	273 (22%)	0.001
Right SBP (mmHg)	106.23 ± 10. 6	134.12 ± 13	< 0.001
Right DBP (mmHg)	67.33 ± 7.8	82.95 ± 7.6	< 0.001

*Independent T-test, HTN: Hypertension; MET: Metabolic equivalent of the task; BMI: Body mass index; Right SBP: Right arm second systolic blood pressure; Right DBP: Right arm first diastolic blood pressure

Regarding dietary intake of the participants, patients with HTN had a higher intake of calorie (2549.93 ± 818.39 vs. 2477.77 ± 770.36 kcal/d), total protein (79.98 ± 26.79 vs. 78.14 ± 25.74 g/d), carbohydrate (425.44 ± 144.70 vs. 407.77 ± 135.06 g/d), isoleucine (3.12 ± 1.05 vs. 3.05 ± 1.01 g/d), leucine (5.36 ± 1.82 vs. 3.75 ± 1.33 g/d), valine (3.67 ± 1.23 vs. 3.06 ± 1.01 g/d), and lower intake of fats (64.31 ± 25.06 vs. 64.58 ± 25.06 g/d) and cholesterol (256.17 ± 121.66 vs. 261.19 ± 120.39 mg/d) compared to the participants without HTN (All *P* < 0.01) (Table 2).

**Table 2:** Dietary intake among the participants

	SBP ≤ 140 and/or DBP ≤ 90 (*n* = 2945)	SBP > 140 and/or DBP > 90 (*n* = 1239)	P*
Calorie (kcal/d)	2477.77 ± 770.36	2549.93 ± 818.39	
Protein (g/d)	78.79 ± 0.19	78.44 ± 0.3	0.324
Fat (g/d)	65.1 ± 0.31	63.08 ± 0.48	< 0.001
Carbohydrate (g/d)	411.37 ± 0.70	416.84 ± 1.09	< 0.001
Cholesterol (mg/d)	261.19 ± 120.39	256.17 ± 121.66	
Tryptophan (g/d)	0.80 ± 0.00	0.8 ± 0.00	0.339
Tryptophan (g/d)	2.53 ± 0.01	2.52 ± 0.01	0.349
Isoleucine (g/d)	3.08 ± 0.01	3.06 ± 0.02	0.445
Leucine (g/d)	5.28 ± 0.02	5.26 ± 0.03	0.493
Lysine (g/d)	3.79 ± 0.016	3.75 ± 0.03	0.208
Methionine (g/d)	1.46 ± 0.01	1.45 ± 0.01	0.506
Cysteine (g/d)	1.16 ± 0.00	1.17 ± 0.01	0.455
Phenylalanine (g/d)	3.29 ± 0.01	3.29 ± 0.02	0.726
Tyrosine (g/d)	3.59 ± 1.18	3.67 ± 1.23	0.004
Valine (g/d)	3.63 ± 0.01	3.61 ± 0.02	0.500
Arginine (g/d)	3.63 ± 0.01	3.60 ± 0.02	0.149
Histidine (g/d)	1.75 ± 0.01	1.74 ± 0.01	0.503
Alanine (g/d)	3.10 ± 0.01	3.10 ± 0.02	0.515
Aspartic Acid (g/d)	5.89 ± 0.02	5.86 ± 0.03	0.532
Glutamic Acid (g/d)	17.02 ± 0.06	17.16 ± 0.09	0.170
Glycine (g/d)	2.66 ± 0.01	2.66 ± 0.01	0.628
Proline (g/d)	5.54 ± 0.02	5.59 ± 0.3	0.302
Serine (g/d)	3.46 ± 0.01	3.45 ± 0.02	0.552
Total BCAAs	11.89	12.16	0.148

*Independent T-test, HTN: Hypertension;

Multivariate logistic regression analysis found that HTN had a marginally positive association with total intake of BCAAs and the intake of different types of BCAAs (All *P* < 0.05) (Table 3, Model 1). After adjusting for age and gender, the association between HTN and total intake of BCAAs (OR = 1.03, CI95%: 1.01–1.05, *P* = 0.01), leucine (OR = 1.06, CI95%:1.01–1.11, *P* = 0.01), isoleucine (OR = 1.11, CI95%:1.02–1.19, *P* = 0.01), and valine (OR = 1.09, CI95%:1.02–1.16, *P* = 0.01) remained significant (Model 2). The results did not change after additional adjustments for education, marital status, job, diabetes, physical activity, and BMI (Model 3). The associations disappeared after further adjustments for energy intake, total protein intake, and other amino acids (Model 4).

**Table 3:** The association of hypertension with dietary intake of branch-chain amino acids (BCAAs)

	Total BCAAs (mg/d)		Leucine (mg/d)		Isoleucine (mg/d)		Valine (mg/d)	
	OR (CI95%)	*P*	OR (CI95%)	*P*	OR (CI95%)	*P*	OR (CI95%)	*P*
Model 1	1.02 (1.01–1.04)	0.04	1.04 (1.01–1.08)	0.03	1.07 (1.01–1.14)	0.04	1.06 (1.01–1.12)	0.04
Model 2	1.03 (1.01–1.05)	0.01	1.06 (1.01–1.11)	0.01	1.11 (1.02–1.19)	0.01	1.09 (1.02–1.16)	0.01
Model 3	1.03 (1.01–1.05)	0.01	1.06 (1.02–1.11)	0.01	1.11 (1.03–1.2)	0.01	1.1 (1.02–1.17)	0.01
Model 4	1.01 (0.96–1.04)	0.99	1.01 (0.92–1.09)	0.99	1.01 (0.87–1.16)	0.99	1.00 (0.88–1.13)	0.99

Model 1: Crude, Model 2: Adjusted for age and gender. Model 3: Further adjustments for education, marital status, job, physical activity, BMI, and diabetes. Model 4: Further adjustments for the intake of energy, and other amino acids

## Discussion

The results of the present study indicated that participants with HTN had a higher intake of total protein and BCAAs than participants with normal BP. Moreover, there was a significant positive association between BP and higher dietary intake of BCAAs. However, the association disappeared after the adjustment of the total protein and calorie intake. Higher intake of BCAAs may increase the risk of HTN through increased consumption of dietary proteins. The present study’s results regarding the association between BCAAs and high BP are consistent with some previous studies [21, 32–36]. For example, Teymoori et al. in a population-based prospective study on 4288 adults reported a positive relationship between the dietary amino acids including BCAAs and increased risk of HTN [32]. Two Asian studies also reported similar findings of a positive relationship between BCAAs and Aromatic Amino Acids (AAAs) with HTN [35, 36]. The ratio of the intake of different types of amino acids can also influence the risk of HTN. Teymoori et al. in a recent study on the ratio of different dietary amino acids found that the Leucine–Serine/Threonine–Tryptophan ratio was positively associated with the risk of HTN [33].

Moreover, the observation of previous studies that high levels of circulating BCAAs are positively associated with increased risk of HTN is in line with our results [23, 37–39]. On the other hand, animal proteins as rich sources of BCAAs are frequently reported to be associated with HTN [40–42]. Nevertheless, in contrast with the results of this study, Jennings et al., in the Twin UK cohort study, found that higher intakes of BCAAs were associated with decreased risk of HTN [43]. Moreover, a Chinese study observed no association between HTN and serum BCAAs levels [44]. A possible cause of these contradictory results is that the effects of BCAAs on BP may be influenced by the total amount of dietary protein, calorie intake, and the level of other nutrients. For example, whey protein is rich in both lactokinins and BCAAs, the first of which has inhibitory properties on the angiotensin-converting enzyme and BCAAs may have an adverse effect on BP; thus, whey protein intake may have both positive and negative impact on hypertension [45].

The exact mechanism of the effect of BCAAs on BP is not clear. Various biochemical mechanisms may explain the observed findings of the impact of BCAAs on BP. It has been shown that alterations in BCAAs metabolism, leading to the aggregation of BCAAs and their byproducts, are linked to striking metabolic derangements such as insulin resistance, the latter being related to an increased risk of HTN [23, 24]. The accumulation of BCAAs and their byproducts can cause impairments in the function of the mammalian target of rapamycin complex 1 (mTORC1) and overstimulation of adenosine monophosphate-activated protein kinase (AMPK), which subsequently leads to insulin resistance and plays a key role in the development of HTN. Also, AAAs and BCAAs have hydrophobic residues that can be relevant to binding bioactive peptides to the angiotensin-converting enzyme, which is crucial in controlling BP [46].

Moreover, high serum levels of serine and BCAAs can play a role in HTN by reducing the entry of threonine, tryptophan, and glutamic acid into the brain, consequently diminishing the synthesis of BP-beneficial neurotransmitters [47, 48]. For example, BCAAs reduce tyrosine and tryptophan transport and serotonin synthesis [48] by competing with tryptophan for entrance to the brain [49]. Serotonin is the primary metabolite of tryptophan, which has a vasodilatory effect through nitric oxide (NO) production [50]. Decreased or impaired serotonin synthesis has been reported in patients with HTN, and a previous study reported a link between increased BP and decreased serotonin synthesis [51]. BCAAs (especially leucine) and their metabolites can also affect BP through adverse effects on insulin sensitivity [52]. Indeed, salt absorption in the proximal tubule is enhanced in humans with insulin resistance due to the preservation of the stimulatory effect of insulin (compensatory hyperinsulinemia) on salt reabsorption in the kidney proximal tubule, resulting in a state of salt overload and HTN [24, 52].

The strengths of current study were a large sample size within a population-based study, allowing generalization of findings to the whole population. However, this study had some limitations. First, despite adjusting for a wide range of variables, the effect of some unknown and unmeasured confounders may have remained. Second, the lack of analysis of the serum BCAAs concentrations and reliance on dietary intake of BCAAs ignores essential factors such as the levels of protein cleavage and amino acid absorption in the gut lumen. Third, the utilization of an FFQ for dietary intake assessment constitutes a limitation due to potential recall bias and the inherent inaccuracies associated with self-reported dietary data, which can compromise the reliability and validity of the findings. More longitudinal studies are needed to confirm the results of the present study.

## Conclusion

In conclusion, the provided data suggest that higher intakes of BCAAs are associated with a higher risk of HTN. If these results are confirmed, a lower intake of BCAAs-rich foods can be considered as a dietary recommendation against HTN. Further research based on direct measurements of amino acids in plasma should be prompted to achieve better and more realistic findings. Clinical trial studies are also needed to confirm these findings and determine the interplay between amino acid metabolism and HTN and whether a high level of these amino acids affects the risk of chronic diseases, especially high BP.

## Data Availability

Datasets used and analyzed during the current study are available from the corresponding author on reasonable requests.
